# Vascular wall regulator of G-protein signalling-1 (RGS-1) is required for angiotensin II–mediated blood pressure control

**DOI:** 10.1016/j.vph.2018.04.002

**Published:** 2018-09

**Authors:** Jyoti Patel, Surawee Chuaiphichai, Gillian Douglas, Caroline M. Gorvin, Keith M. Channon

**Affiliations:** a Division of Cardiovascular Medicine, British Heart Foundation Centre of Research Excellence, Radcliffe Department of Medicine, John Radcliffe Hospital, University of Oxford, Oxford, OX3 9DU, UK; b Wellcome Centre for Human Genetics, University of Oxford, Oxford, OX3 7BN, UK; c Academic Endocrine Unit, Oxford Centre for Diabetes, Endocrinology, and Metabolism, Radcliffe Department of Medicine, University of Oxford, Oxford, OX3 7LE, UK

**Keywords:** Rgs1, G-protein signalling, Angiotensin II, Smooth muscle cell, Vascular function, Rgs1, Regulator of G-protein signalling 1, VSMC, vascular smooth muscle cells, Ang II, angiotensin II, GPCR, G-protein coupled receptor (GPCR)

## Abstract

G-Protein coupled receptors (GPCRs) activate intracellular signalling pathways by coupling to heterotrimeric G-proteins that control many physiological processes including blood pressure homeostasis. The Regulator of G-Protein Signalling-1 (RGS1) controls the magnitude and duration of downstream GPCR signalling by acting as a GTPase-activating protein for specific Gα-proteins. RGS1 has contrasting roles in haematopoietic and non-haematopoietic cells. *Rgs1*^*−/−*^*ApoE*^*−/−*^ mice are protected from Angiotensin II (Ang II)-induced aortic aneurysm rupture. Conversely, Ang II treatment increases systolic blood pressure to a greater extent in *Rgs1*^*−/−*^*ApoE*^*−/−*^ mice than *ApoE*^*−/−*^ mice, independent of its role in myeloid cells. However the precise role of RGS1 in hypertension and vascular-derived cells remains unknown. We determined the effects of *Rgs1* deletion on vascular function in *ApoE*^*−/−*^ mice. *Rgs1* deletion led to enhanced vasoconstriction in aortas and mesenteric arteries from *ApoE*^*−/−*^ mice in response to phenylephrine (PE) and U46619 respectively. *Rgs1* was shown to have a role in the vasculature, with endothelium-dependent vasodilation being impaired, and endothelium-independent dilatation to SNP being enhanced in *Rgs1*^*−/−*^*ApoE*^*−/−*^ mesenteric arteries. To address the downstream signalling pathways in vascular smooth muscle cells (VSMCs) in response to Ang II-stimulation, we assessed pErk1/2, pJNK and pp38 MAPK activation in VSMCs transiently transfected with Rgs1. pErk1/2 signalling but not pJNK and pp38 signalling was impaired in the presence of Rgs1. Furthermore, we demonstrated that the enhanced contractile response to PE in *Rgs1−/−ApoE−/−* aortas was reduced by a MAPK/Erk (MEK) inhibitor and an L-type voltage gated calcium channel antagonist, suggesting that Erk1/2 signalling and calcium influx are major effectors of Rgs1-mediated vascular contractile responses, respectively. These findings indicate RGS1 is a novel regulator of blood pressure homeostasis and highlight RGS1-controlled signalling pathways in the vasculature that may be new drug development targets for hypertension.

## Graphical abstract

**Figure:**
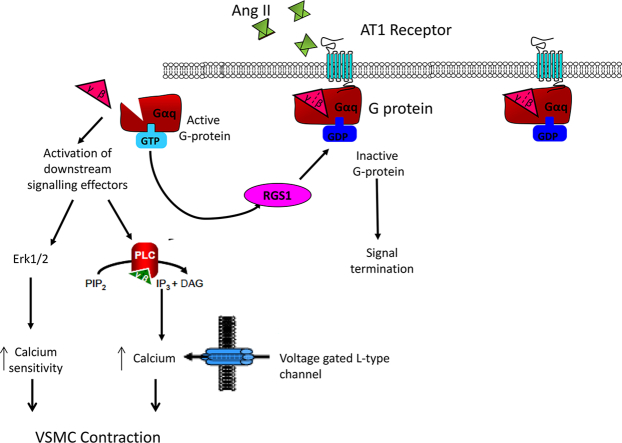

## 1 Introduction

Hypertension is a key risk factor for the development of cardiovascular diseases that can be associated with increased G protein-coupled receptor (GPCR) signalling. Vasoactive substances that activate GPCRs can regulate blood pressure homeostasis by effecting vessel tone acutely. However, longer term changes in systemic pressure and cardiac output leading to vascular dysfunction can result in clinical complications, indicating that tight regulation of GPCR signalling pathways is required to control blood pressure [1]. Many physiologically important vasoactive substances such as angiotensin II (Ang II), noradrenaline, acetylcholine (ACh) signal through multiple GPCRs that couple to the heterotrimeric G-protein families of the Gαq and Gαi subunits [2]. Vascular smooth muscle cells (VSMCs) are responsible for the contractile and dilatory activity of the vessel wall and thereby modulate peripheral resistance [3]. In SMCs, both Ang II and noradrenaline mediate vasoconstriction by activation of Gαq coupled receptors. Signalling by Gαq coupled receptor signalling activates several intracellular pathways including diacylglycerol (DAG) and inositol 1,4,5-trisphosphate (IP_3_), resulting in activation of protein kinase C (PKC) and calcium release from intracellular stores, respectively [4]. Calcium entry across the plasma membrane and release from intracellular stores consequently leads to myosin light chain (MLC) phosphorylation, activation and ultimately vasoconstriction.

Regulator of G-Protein Signalling (RGS) proteins can modulate G-protein signalling by acting as GTPase-activating proteins (GAPs) by accelerating the intrinsic GTPase activity of the Gα subunit leading to reformation and inactivation of the heterotrimer [5]. By promoting G-protein inactivation, the intracellular response to repeated ligand stimulation is down regulated. We have previously shown that RGS1 has a critical role in regulating Ang II mediated aortic aneurysm rupture in *ApoE*^*−/−*^ mice through attenuation of Gαi chemokine receptor signalling in leukocytes [6]. *Rgs1* expression is up-regulated in activated inflammatory cells where it functions to inhibit migration, thus promoting the accumulation of leukocytes during inflammation. Furthermore, there is indication that RGS1 is involved in Ang II-mediated blood pressure control via Gαq signalling, independent of its role in myeloid cells and aneurysm formation. Other RGS proteins have been implicated in blood pressure control [1], however, to date, the role of RGS1 in non-haematopoetic cells and as a regulator of vascular tone has not been explored. Understanding the mechanisms that underlie G-protein signalling pathways in blood pressure homeostasis may provide new therapeutic targets for hypertension.

To identify physiological mechanisms by which RGS1 regulates blood pressure, we investigated the role of *Rgs1* in vessel wall activation, and the effect of *Rgs1* deletion on vascular function in the conduit and resistance vasculature. We used *Rgs1*^*−/−*^ mice on an *ApoE*^*−/−*^ background, as hyperlipidemia is a common co-morbidity with hypertension. Our findings provide the first evidence indicating that RGS1 mediates blood pressure by promoting relaxation of the resistance vasculature through its ability to attenuate vasoconstrictor-induced signalling.

## 2 Methods

### 2.1 Mice

The generation of *Rgs1*^*−/−*^ mice has been previously described [7]. *Rgs1*^*−/−*^ mice were crossed onto an *ApoE*^*−/−*^ background (Charles River, UK) to generate matched litters of *Rgs1*^*−/−*^*ApoE*^*−/−*^ and *ApoE*^*−/−*^ mice. Mice were housed in individually ventilated cages with 12 h light/dark cycle and controlled temperature (20 °C - 22 °C). Standard chow (B & K Universal Ltd., UK) and water were available ad libitum. All animal studies were conducted with ethical approval from the Local Ethical Review Committee and in accordance with the UK Home Office Animals (Scientific Procedures) Act 1986. For bone marrow transplantation studies, ten-week-old male mice received a lethal dose of whole-body irradiation (2 × 5 Gy) followed by an i.v. injection of 5 × 10^6^ bone marrow cells from male *Rgs1*^*−/−*^*ApoE*^*−/−*^ and *ApoE*^*−/−*^ mice.

### 2.2 Quantitative real time RT-PCR

Total RNA was isolated using RNeasy kits (Qiagen, UK) and reverse transcribed to cDNA using SuperScript II Reverse Transcriptase (Invitrogen, UK). Quantitative real-time PCR was performed with 10–50 ng of cDNA on an iCycler IQ real time detection system (Bio-Rad Laboratories Ltd., UK). Gene expression was determined using TaqMan Gene Expression Assays (Applied Biosystems, UK) relative to the level of the house keeping genes β-actin for mouse, and GAPDH for human using real time RT-PCR. Relative quantitation of gene expression was performed using the comparative Ct method (ΔΔCt).

### 2.3 Primary cell isolation, culture and cell line transfections

Primary cells and whole thoracic aortas were obtained from 16-week-old *ApoE*^*−/−*^ and *Rgs1*^*−/−*^*ApoE*^*−/−*^ mice. Primary endothelial cells were isolated from PBS perfused lung tissue. Lungs were finely minced and digested in an enzyme solution of DMEM containing 0.18 U/ml Liberase Blendzyme 3 (130 μl; Roche, UK) and 0.1 mg/ml DNase I (100 μl, Roche, UK) for 1 h at 37 °C with gentle agitation. Purification of endothelial cells was performed by magnetic cell sorting (Miltenyi Biotec, UK) by positive selection using anti-CD31 microbeads (Miltenyi Biotec, UK). Primary VSMCs were isolated by the aortic explant method. Aortas were cut into small segments following endothelial cell denudation and adhered onto 2% gelatin coated wells and cultured in DMEM complete growth media for 2 weeks. Outgrowing VSMCs from explants of aortic tissue were harvested using Trypsin/EDTA and pelleted for RNA.

The murine vascular aortic smooth muscle cell line, MOVAS (ATCC CRL-2797) were transiently transfected at 50% confluency with plasmid expressing Myc-DDK (FLAG) tagged murine Rgs1 (Origene) or an empty mammalian expression vector (pcDNA3.1) using FuGENE HD (Roche) in serum free DMEM media. Transfections were performed at 3:1 ratio of FuGENE HD to 1 μg DNA. To check for transfection efficiency, in parallel experiments, intracellular staining and flow cytometry was performed for the FLAG tag using an anti-FLAG FITC antibody (Sigma). For staining of intracellular FLAG expression, cells were washed, fixed in 2% PFA (Life Technologies,) then permeabilised by incubation with Cytofix/Cytoperm then washed in PermWash (BD Biosciences). Cells were then incubated in Fc block and antibody stained for FLAG. Data were acquired using a CyAn Analyser flow cytometer (Beckman Coulter, UK) and then analysed using Summit (Dako, UK) and FlowJo (Tree Star Inc., USA) software. Between 48 and 72 h, the time point at which the signalling assays were performed, 72% of all cells were FLAG tag positive, indicating RGS1 expression in transfected cells (Supplementary Fig. 1).

### 2.4 Measurement of pErk, pJNK and pp38 MAPK

MOVAS cells were seeded in 24-well plates and transfected with pcDNA3.1 or *Rgs1*, 24 h prior to performance of assays. Transfected cells were incubated in serum-free media 12 h prior to treatment of cells with 1 uM of Angiotensin II over 30 min. Cells were then lysed in Surefire lysis buffer, and AlphaScreen Surefire pERK, pJNK, pp38, total ERK and GAPDH assays (PerkinElmer, Beaconsfield, UK) measuring phosphorylated and total proteins were performed, as described [8]. The fluorescence signal in both assays was measured using the PHERAstar FS microplate reader (BMG Labtech). The ratio of phosphorylated ERK (pERK) to total ERK, pJNK and pp38 to GAPDH was measured at each time point. All assay conditions were performed in four biological replicates on two independent occasions.

### 2.5 Angiotensin II infusion and unconscious arterial pressure monitoring

16 week old chimeric male mice were anaesthetized with isoflurane by inhalation and osmotic mini pumps (Alza Corp, USA) delivering Ang II (3 mg kg^−1^ per day; Sigma-Aldrich) for 14 days were implanted subcutaneously. At day 14, mean arterial blood pressure and heart rate was measured by catheterizing the right common carotid artery with a Millar 1.4 French blood pressure probe and transducer (Millar Instruments Inc., Houston, TX) coupled to a Powerlab/4sp data acquisition system (ADInstruments Pty Ltd., New Castle, Australia) and passing the probe into the ascending aorta. After 15 min equilibrium period blood pressure was measured for 5 min and the average recordings for this time taken.

### 2.6 Isometric tension vasomotor studies

Vasomotor function was analysed using isometric tension studies in a wire myograph (Multi-Myograph 610 M, Danish Myo Technology, Denmark). Briefly, mice were culled by overdose of inhaled isoflurane and vascular rings were isolated from the thoracic aorta or mesenteric arcades. The aortic rings or 2nd order mesenteric arteries (2 mm) were mounted on a wire myograph containing 5 ml of Krebs–Henseleit buffer (KHB [in mmol/l]: NaCl 120, KCl 4.7, MgSO_4_ 1.2, KH_2_PO_4_ 1.2, CaCl_2_ 2.5, NaHCO_3_ 25, glucose 5.5) at 37 °C, gassed with 95% O2/5% CO2. After allowing vessels to equilibrate for 30 min, the optimal tension was set (equivalent to 100 mmHg). The vessel viability was tested using 60 mM KCl. Concentration–response contraction curves were established using cumulative half-log concentrations of phenylephrine, U46619 and Ang II. Acetylcholine and SLIGRL were used to stimulate endothelium-dependent vasodilatations in increasing cumulative concentrations. Responses were expressed as a percentage of the pre-constricted tension. The NO donor sodium nitroprusside (SNP) was used to test endothelium-independent smooth muscle relaxation in the presence of 100 μM L-NAME. The MEK inhibitor, PD98059 (10 uM) and the L-type voltage gated calcium channel antagonist, Nifedipine (50 nM) were used to block PE induced vasoconstriction in aortas. All pharmacological drugs were pre-incubated at least 20 min before the dose–response curves were determined. All drugs used were purchased from Sigma Aldrich.

### 2.7 Statistical analysis

Between group comparisons of normally distributed measurements were assessed by Student's *t*-test. One-way AVOVA was used to compare more than two data groups and Dunnett's post-test was used to compare each group to a control (untreated) group. Two-way ANOVA was used to compare multiple data groups affected by two independent variables, with a Bonferroni correction to compare groups with each other. Differences were considered statistically significant at P < 0.05.

## 3 Results

### 3.1 *Rgs1* deficiency in non-haematopoietic cells results in Angiotensin II- induced hypertension in *ApoE*^*−/−*^ mice

**Fig. 1:**
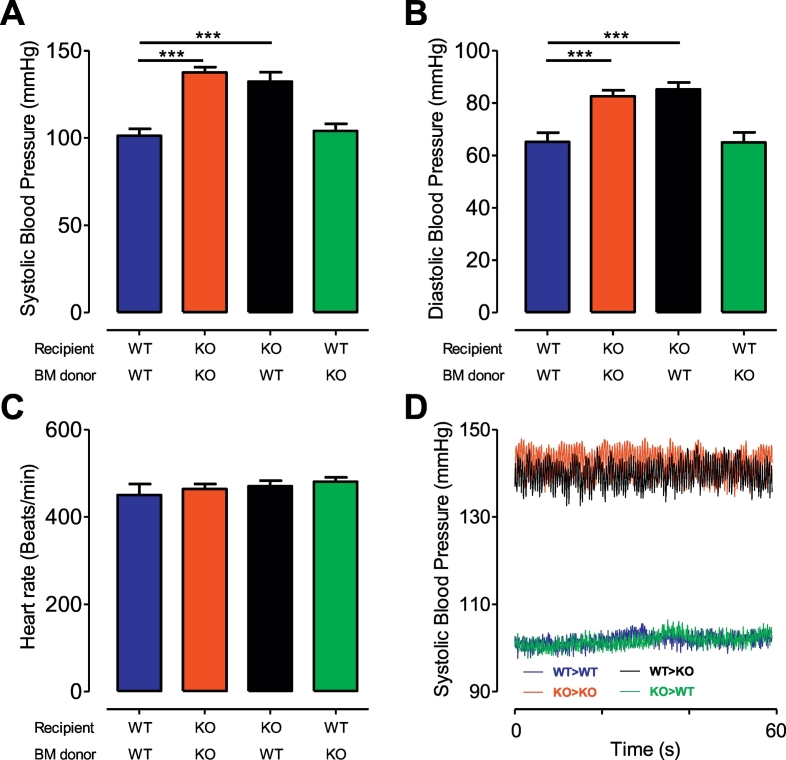
Rgs1−/−ApoE−/− chimeric mice have increased blood pressure responses after Ang II infusion for 14 days. Chimeric *ApoE*^*−/−*^ and *Rgs1*^*−/−*^*ApoE*^*−/−*^ mice were infused with Ang II (3 mg kg^−1^ per day) over 14 days and blood pressure measured at day 14 in anaesthetized mice using a Millar catheter. (A) Systolic blood pressure (B) Diastolic blood pressure (C) Heart rate in Rgs1−/−ApoE−/− and ApoE−/− chimeric mice. (D) Representative traces of systolic blood pressure in chimeric mice using a Millar catheter (***P < 0.001; n = 10–11 animals per group). WT = ApoE−/−; KO = Rgs1−/−ApoE−/−.

We had previously shown that Ang II infusion for 14 days resulted in a significant increase in blood pressure in *Rgs1*^*−/−*^*ApoE*^*−/−*^ mice. We first aimed to establish if the increase in blood pressure that was observed at the slow pressor dose used previously, was still present at a higher pressor dose of Ang II. Invasive blood pressure recordings in unconscious mice were performed after 14 days of Ang II infusion (3 mg/kg/day). Consistent with our previous study [6], we found significant differences in systolic blood pressure between *ApoE*^*−/−*^ mice transplanted with *ApoE*^*−/−*^ bone marrow and *Rgs1*^*−/−*^*ApoE*^*−/−*^ mice transplanted with *Rgs1*^*−/−*^*ApoE*^*−/−*^ bone marrow (101.3 ± 3.944 mmHg versus 137.5 ± 3.056 mmHg; *P* < 0.0001; Fig. 1A). In addition an increase in systolic blood pressure was also observed in *Rgs1*^*−/−*^*ApoE*^*−/−*^ mice transplanted with *ApoE*^*−/−*^ bone marrow (101.3 ± 3.944 mmHg versus 132.4 ± 5.218 mmHg; P = 0.0002; Fig. 1A, D), indicating that the increase in systolic blood pressure was not due to a contribution of haematopoietic cells as described in previous studies [9]. In line with the changes observed in systolic blood pressure, a significant increase in diastolic blood pressure between *ApoE*^*−/−*^ mice transplanted with *ApoE*^*−/−*^ bone marrow and *Rgs1*^*−/−*^*ApoE*^*−/−*^ mice transplanted with *ApoE*^*−/−*^ bone marrow was significantly altered (65.18 ± 3.462 mmHg versus 85.21 ± 2.652 mmHg; Fig. 1B; P = 0.0002). Heart rate was similar between *ApoE*^*−/−*^ and *Rgs1*^*−/−*^*ApoE*^*−/−*^ mice (450.2 ± 25.29 beats/min versus 470.2 ± 13.06 beats/min; Fig. 1C).

### 3.2 *Rgs1* mRNA down-regulation in response to prolonged Ang II stimulation

**Fig. 2:**
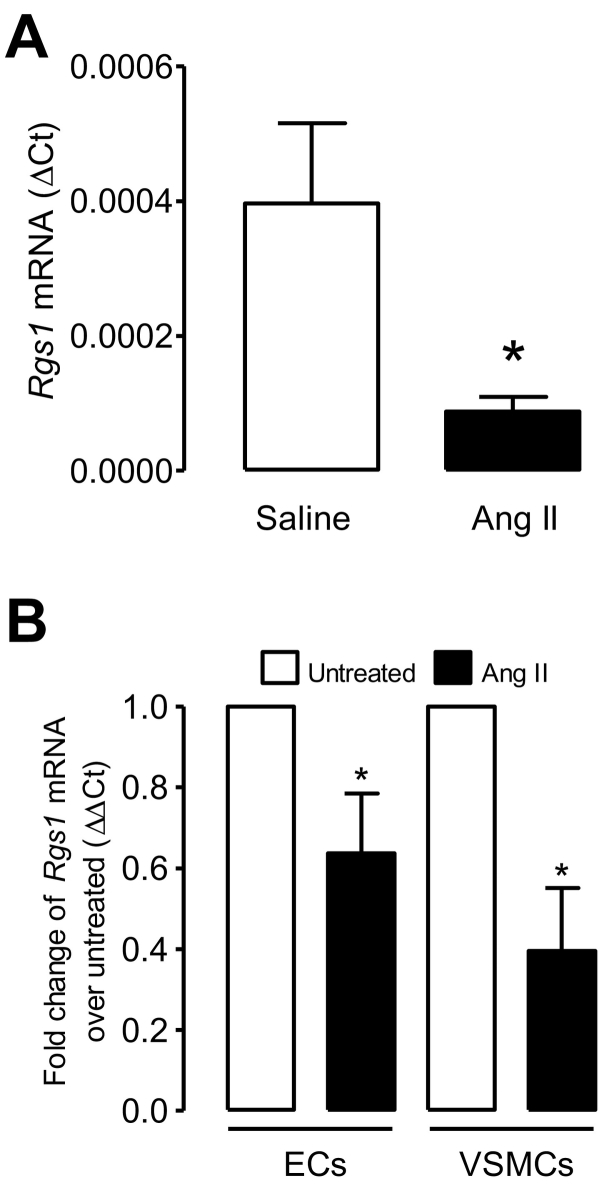
Rgs1 mRNA is down-regulated following Ang II stimulation. qRT–PCR analysis of *Rgs1* mRNA in (A) thoracic aortas from *ApoE*^*−/−*^ mice following 5 day saline or Ang II infusion (0.8 mg/kg/day) (B) primary endothelial cells and primary VSMCs from *ApoE*^*−/−*^ mice stimulated for 24 h with Ang II (1 uM) in vitro after a 5 day culture presented relative to mRNA in unstimulated cells, set as 1 (n = 4–7; *P < 0.05).

We next examined the possible regulation of *Rgs1* to the effects of Ang II-mediated AT1 receptor activation in the vasculature. To assess this we measured the expression of *Rgs1* following the administration of Ang II in vivo and in vitro. We first measured the expression of *Rgs1* mRNA in thoracic aortas from *ApoE*^*−/−*^ mice implanted with saline and Ang II for 5 days and found *Rgs1* mRNA expression was significantly reduced in aortas from Ang II-treated *ApoE*^*−/−*^ mice compared to saline treated mice (Fig. 2A). We next measured expression in isolated and cultured primary endothelial and VSMCs stimulated with Ang II in vitro (Fig. 2B) and found *Rgs1* expression was reduced with stimulation. These findings indicate RGS1 is a likely candidate for negative regulation of the Gαq proteins coupled to the Ang II type 1 receptor in the vascular wall, and endothelial and VSMCs.

### 3.3 *Rgs1* deletion leads to enhanced vasoconstriction in aortas and mesenteric arteries from *ApoE*^*−/−*^ mice

VSMCs express a number of GPCRs, such as adrenergic receptors that are essential in mediating sympathetic signalling. Alpha-adrenergic receptors are Gαq coupled and induce phospholipase C (PLC) activation and a subsequent increase in intracellular calcium which is required for the initiation of calcium dependant SMC contraction, and in vessels; calcium dependant vasoconstriction.

**Fig. 3:**
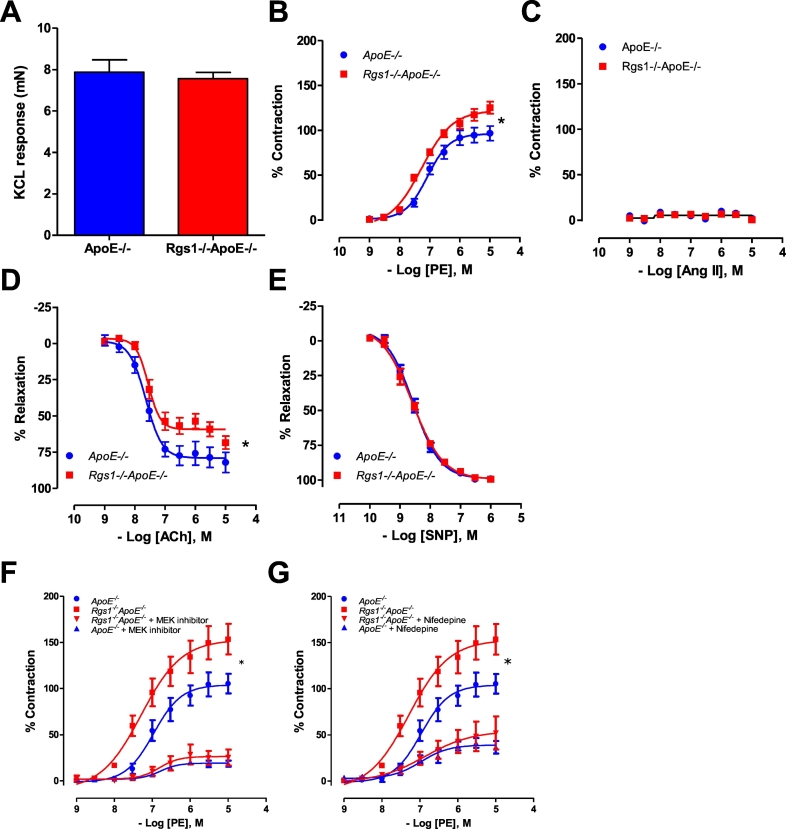
*Rgs1−/−ApoE−/−* aortas have altered vasoconstrictor and vasodilator responses. (A) Maximal contraction of aortas to 45 mmol·L^−1^ KCl. (B) Vasoconstriction to phenylephrine (PE) was enhanced in Rgs1−/−ApoE−/− aortas when compared with *ApoE−/−* aortas. (C) Ang II did not cause a contractile response of the aorta in either group. (D) Endothelial-dependent vasodilatation to acetylcholine (Ach) was altered in *Rgs1−/−ApoE−/−* aortas and (E) Endothelium-independent vasodilatation in response to sodium nitroprusside (SNP) was unchanged between groups (F) Vasoconstriction to PE was abolished in the presence of a MEK inhibitor (10 uM PD98059) in both *Rgs1−/−ApoE−/−* and *ApoE−/−* aortas. (G) Vasoconstriction to PE was partially blunted in the presence of a L-type voltage gated calcium channel antagonist (50 nM Nifedipine) in both *Rgs1−/−ApoE−/−* and *ApoE−/−* aortas (*P < 0.05; A-E; n = 6 animals per group F-G n = 4 animals per group).

To test the effect of RGS1 in Gαq vasomotor function, we determined vascular reactivity in conduit vessels from *ApoE*^*−/−*^ and *Rgs1*^*−/−*^*ApoE*^*−/−*^ mice. No difference was detected between the contraction response to 60 mmol∙L^−1^ KCl in *ApoE*^*−/−*^ (7.872 ± 0.59 mN mm^−1^) and *Rgs1*^*−/−*^*ApoE*^*−/−*^ aortas (7.561 ± 0.30 mN mm^−1^) (Fig. 3A). Receptor-mediated vasoconstrictions to α-adrenergic agonist, phenylephrine (PE), were significantly enhanced in *Rgs1*^*−/−*^*ApoE*^*−/−*^ aortas compared to *ApoE*^*−/−*^ controls (Fig. 3B). In contrast to PE, receptor-mediated vasoconstrictions to AT1 agonist, Ang II had very little effect on vasoconstrictions in aortas from both *ApoE*^*−/−*^ and *Rgs1*^*−/−*^*ApoE*^*−/−*^ mice (Fig. 3C). Additionally, endothelium-dependent vasodilatation was impaired in *Rgs1*^*−/−*^*ApoE*^*−/−*^ aortas compared to *ApoE*^*−/−*^ control aortas (Fig. 3D), but there was no difference in endothelium-independent vasodilatation to SNP between the genotypes (Fig. 3E). To address the finding that the enhanced contractile response to vasoconstrictors in Rgs1−/−ApoE−/− aortas was in part due to MAPK signalling we assessed vasoconstriction to PE in the presence of a MAPK/Erk (MEK) inhibitor and found the response was blunted (Fig. 3F). Smooth muscle contraction is also dependent on calcium signalling, therefore we assessed the response of Rgs1−/−ApoE−/− aortas to PE vasoconstriction in the presence of a L-type voltage gated calcium channel antagonist. We observed that the enhanced contractile response to PE in Rgs1−/−ApoE−/− aortas was reduced by an L-type voltage gated calcium channel antagonist to the same extent as that of ApoE−/− aortas(Fig. 3G). These observations indicate that calcium signalling and MAPK signalling are major effectors of Rgs1-mediated vascular contractile responses, which are known to be activated downstream of the Gaq receptor signalling.

**Fig. 4:**
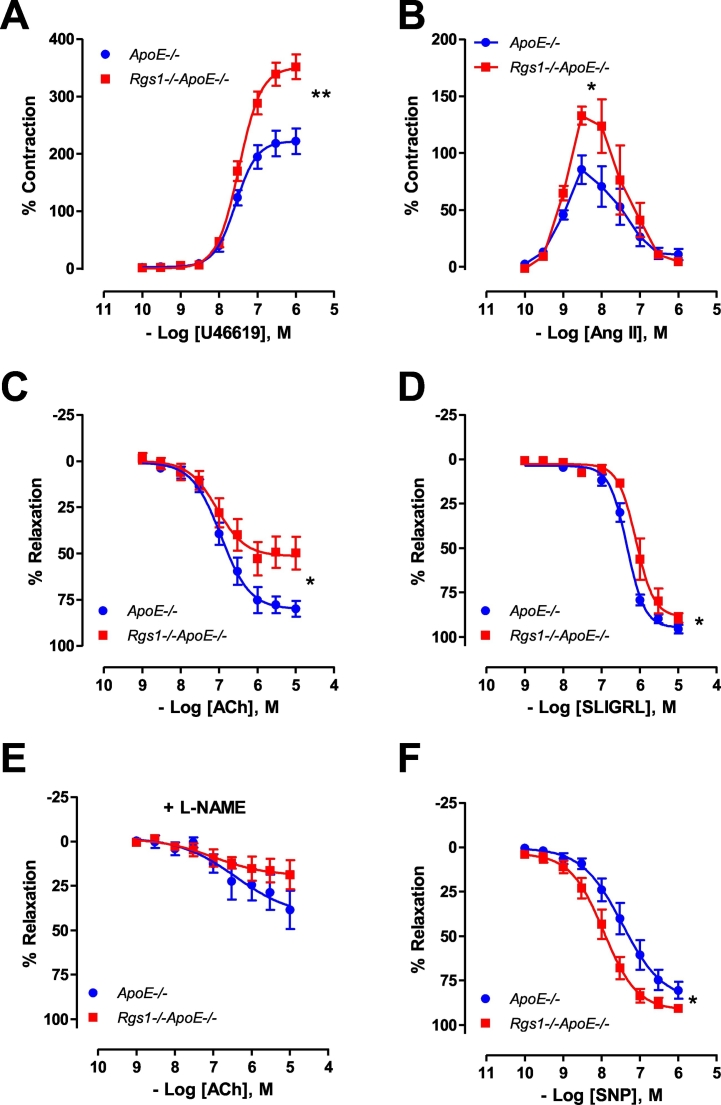
Altered vasomotor function in isolated 2nd order mesenteric arteries from Rgs1−/−ApoE−/− mice. Vasoconstriction in responses to (A) U46619 and (B) Ang II was enhanced in 2nd order mesenteric arteries from Rgs1−/−ApoE−/− mice compared to ApoE−/− mice. Endothelium-dependent vasodilatation to (C) acetylcholine (Ach) and (D) SLIGRL were impaired in Rgs1−/−ApoE−/− mesenteric arteries. (E) The vasodilatory response to ACh were normalised in the presence of non-selective nitric oxide synthase inhibitor (100 mM; L-NAME) and showed no change between groups. (F) Endothelium-independent vasodilatation to sodium nitroprusside (SNP) was impaired in ApoE−/− mesenteric arteries compared to Rgs1−/−ApoE−/− mesenteric arteries (*P < 0.05, **P < 0.01; n = 6 to 9 animals per group).

To further investigate the relationships between blood pressure and changes in the resistance vasculature, mesenteric arteries from *ApoE*^*−/−*^ and *Rgs1*^*−/−*^*ApoE*^*−/−*^ mice were isolated and the contractile response to vasoconstrictors and vasodilators were determined. In *Rgs1*^*−/−*^*ApoE*^*−/−*^ mesenteric arteries, receptor-mediated vasoconstrictions to thromboxane A_2_ receptor agonist, U46619, were significantly enhanced compared to *ApoE*^*−/−*^ controls (Fig. 4A). In addition, receptor-mediated vasoconstrictions to AT1 receptor agonist, Ang II were also markedly enhanced in *Rgs1*^*−/−*^*ApoE*^*−/−*^ compared to *ApoE*^*−/−*^ (Fig. 4B). Endothelium-dependent vasodilatations in response to ACh and the proteinase-activated receptor 2 agonist, SLIGRL were both significantly impaired in *Rgs1*^*−/−*^*ApoE*^*−/−*^ mesenteric arteries when compared with that in *ApoE*^*−/−*^ mesenteric arteries (Fig. 4C, D). The vasodilatory response to ACh were normalised in the presence of non-selective nitric oxide synthase inhibitor (L-NAME) and showed no change between groups (Fig. 4E). Furthermore, endothelium-independent vasodilatation to SNP was enhanced in *Rgs1*^*−/−*^*ApoE*^*−/−*^ mesenteric arteries when compared to mesenteric arteries from *ApoE*^*−/−*^ mice (Fig. 4F), indicating the impairment of vasodilatation in *Rgs1*^*−/−*^*ApoE*^*−/−*^ mesenteric arteries was due to the alteration of vascular smooth muscle sensitivity. Together, these findings indicate that *Rgs1* regulates blood pressure in resistance arteries.

### 3.4 *Rgs1* attenuates Ang II stimulated Erk1/2 phosphorylation in VSMCs

**Fig. 5:**
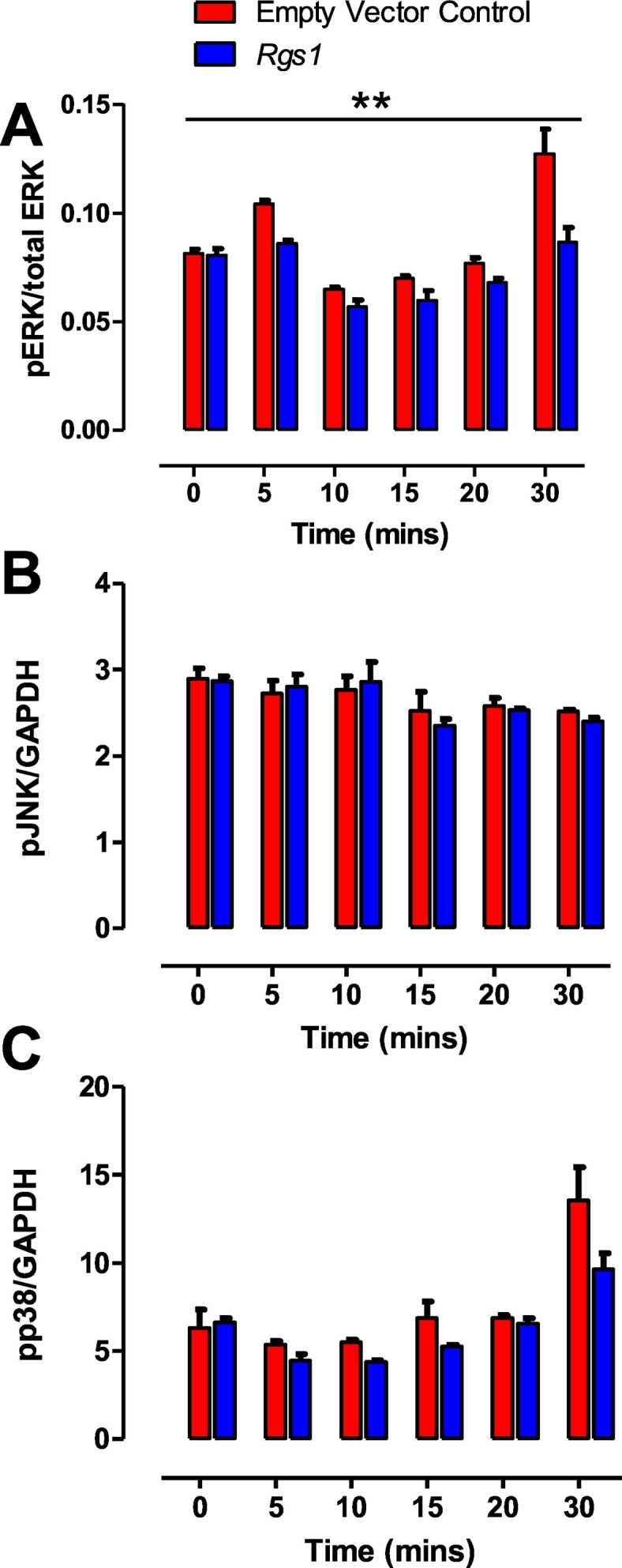
*Rgs1* reduces Ang II activation of pErk1/2 signalling in VSMCs. (A) Rgs1 transfected VSMCs have reduced pErk1/2 activation following 1 μM Ang II stimulation over 30 min but not (B) pJNK and (C) pp38 activation (**P < 0.01; n = 4 technical replicates, 2 independent experiments).

Ang II signals through AT1 receptors (AT1R), that are implicated in AngII-induced vasoconstriction. Following activation of the receptor, Gα and Gβγ subunits dissociate, activating downstream signalling cascades. Extracellular regulated kinases 1 and 2 are ubiquitously utilized proteins for examining vasoactive signalling due to their role in the propagation of ligand activated signalling through GPCRs [10]. Specifically, this signalling has been connected to Gαq and Gαi signalling, and can lead to a number of intracellular downstream effects ranging from migration and adhesion to proliferation. ERK is phosphorylated and activated by at least three downstream AT1R signalling pathways, including the protein kinase C (PKC)-dependent pathway, the β-arrestin-dependent pathway, and the epidermal growth factor receptor (EGFR) transactivation pathway [11]. To assess the role of RGS1 in the Ang II-stimulated activation of the MAPK signalling pathway, we assessed the levels of phosphorylated Erk1/2 protein and other known kinases induced by Ang II, using transient transfections of Rgs1 in the murine vascular smooth muscle cell line (MOVAS). We found that RGS1 reduced pERK signalling following Angiotensin II stimulation over a 30 min time course (Fig. 5A), but not pJNK or pp38 signalling (Fig. 5B, C). Furthermore, we also found pERK levels at 10, 15 and 20 min were below baseline. The mechanisms underlying the increased contraction in Rgs1 deficient vessels and Ang II-mediated hypertension in Rgs1−/−ApoE−/− mice are likely to involve different signalling cascades involving both MAPK signalling and calcium influx, which are known to be activated downstream of the alpha-adrenergic receptor and AT1R. Numerous signalling pathways in response to Ang II are mediated by reactive oxygen species (ROS) and oxidative stress underlies major vascular diseases including atherosclerosis and abdominal aortic aneurysm. Ang II-induced ROS including superoxide and hydrogen peroxide, can influence vascular tone, remodelling and induce hypertension by vascular constriction, as well as nitric oxide inactivation [12]. We assessed hydrogen peroxide released from VSMC transfected with Rgs1, stimulated with Ang II for 12 h. We observed no differences in basal hydrogen peroxide production, and after Ang II stimulation in supernatants from VSMCs transfected with Rgs1 (data not shown).

## 4 Discussion

The modulation of blood pressure by GPCRs is essential in the maintenance of normal cardiovascular function, which when compromised can result in vascular dysfunction and hypertension, a risk factor for peripheral atherosclerosis, renal failure, myocardial infarction and stroke. Furthermore, GPCR activity has been a target by many drugs to treat hypertension, including AT1 receptor and β- adrenergic receptor antagonists [13]. To date, three members of the RGS protein family; RGS2, RGS4 and RGS5 have been linked to blood pressure maintenance [1]. Our results provide new insights into the role of RGS1 as an additional regulator of vascular tone and blood pressure. In this study we have shown that loss of *Rgs1* on the hyperlipidemic ApoE−/− background resulted in an enhanced arterial constrictor response in both conduit and resistance arteries. In addition, the loss of Rgs1 resulted in endothelial cell dysfunction with a blunted endothelial dilator response, which was apparent despite an increase in VSMC sensitivity to NO.

Hypertension can be associated with the dysfunction of the renin angiotensin system (RAS) [14]. Angiotensin II (Ang II), a primary effector of the RAS, is produced both systemically and locally in the vessel wall [15] with its effects mediated by the Angiotensin type I receptors. We show that loss of Rgs1 leads to an increased sensitivity to chronic treatment with Ang II resulting in a significant increase in both systolic and diastolic blood pressure. Previous studies have shown a role of haematopoietic cells, specifically T cells in the hypertensive response to Ang II [9]. Using the *rag1−/−* mice lacking T and B cells and adoptive transfer, Guzik et al., demonstrated hypertension was associated with T-cell infiltration in perivascular tissue, oxidative stress and impairment in endothelial-dependent vasodilatation. As we and others have previously shown, RGS1 is highly expressed in leukocytes and plays a critical role in myeloid cell [6] and lymphoid cell [16] retention in inflammatory disease pathogenesis. Hence in order to ascertain if the increased blood pressure response in *Rgs1*^*−/−*^*ApoE*^*−/−*^ Ang II treated mice was in part due to a contribution of RGS1 from leukocytes we generated bone marrow chimeras. The increased blood pressure response was only observed in *Rgs1*^*−/−*^*ApoE*^*−/−*^ receiving *Rgs1*^*−/−*^*ApoE*^*−/−*^ or *ApoE*^*−/−*^ bone marrow, strongly implicating loss of Rgs1 in the vessel wall, results in the enhanced hypertensive response to Ang II. In our previous study, we examined the T-cell and B-cell phenotype of *Rgs1*^*−/−*^*ApoE*^*−/−*^ mice and found no significant alterations, suggesting RGS1 in lymphocytes in the context of atherosclerosis and Ang II induced AAA has a less significant role to that in myeloid cells in vascular inflammation. We did not observe any differences in heart rate between *Rgs1*^*−/−*^*ApoE*^*−/−*^ mice and *ApoE*^*−/−*^ mice despite the observed increase in blood pressure. Previous studies have demonstrated that Ang II modifies the barrow receptor reflex either by decreasing sensitivity, or by resetting the reflex to a higher value [17,18], and this central effect most likely explains the absence of reflex bradycardia. Other RGS proteins have been implicated in the aetiology of hypertension. There is strong evidence linking *Rgs2* and hypertension in humans. Genetic studies have identified *Rgs2* single nucleotide polymorphisms (SNPs) in hypertension cohorts from several regions around the world [19]. *Rgs1* also resides in the same locus of chromosome 1q, which harbours multiple susceptibility genes that affect blood pressure levels [20,21], indicating potential SNPs in *Rgs1* could also affect hypertension in humans. Previous studies have shown that *Rgs2*^*−/−*^ mice are hypertensive at baseline and following Ang II infusion [22]. RGS2 is required for cGMP-mediated inhibition of vasoconstrictor-triggered Ca2+ signalling [23,24].

The loss of RGS1 resulted in a significant increase in the contractile response to phenylephrine in the aorta, and to Ang II and the thromboxane mimetic U46619 in resistance arteries. It is unlikely that the enhanced constrictor response observed is due to an alteration in remodelling in these vessels as there was no difference in the contractile response to KCL. The enhanced contraction to PE was blunted in both Rgs1−/−ApoE−/− mice and ApoE−/− aortas following addition of MEK inhibitor and an L-type voltage gated calcium channel antagonist. Contraction is maintained by calcium sensitisation of the contractile proteins via several signalling pathways. Phenylephrine, Ang II [25] and U46619 [26] act via Gaq signalling to cause Ca2+ dependent phosphorylation of MLC via activation of phospholipase C. In addition these agonists also activate G12/13 signalling which increases calcium sensitivity by Rho kinase-mediated inhibition of MLC phosphorylation. Erk1/2 can potentiate contraction via caldesmon phosphorylation, leading to separation from actin and thereby increasing the binding of actin and myosin [27]. Rgs1 is an upstream effector of many signalling pathways including the MAPK pathway, acting at the level of the Ga protein to cause signal termination. Previous in vitro studies have shown that RGS1 is a Gai and Gαq GTPase-activating protein and a potential G12 effector antagonist [28]. It is likely that the enhanced constrictor response observed in the aorta and mesenteric arteries of *Rgs1*^*−/−*^*ApoE*^*−/−*^ mice is due to loss of RGS1 inactivation of Gq and G12/13 and loss of signal termination. Furthermore, receptor stimulation by Ang II is followed by a rapid desensitization of the intracellular signal transduction and a decrease in cell surface receptor number, which could account for the drop in pErk1/2 levels from baseline following Ang II stimulation of VSMCs. In *Rgs2*^*−/−*^ mice, the enhanced constrictor response was due to loss of the NO mediated upregulation of RGS2 activity, leading to an alteration in balance between the constrictor and dilator responses towards a constricted phenotype. It is unlikely that a similar mechanism is responsible in our study, as in contrast the *Rgs2*^*−/−*^ mice, we observed an enhanced dilatory response to the NO donor SNP. The absence of RGS1, effects the regulation of VSMC contraction and dilation in response to the agonists tested, but does not rule out the possibility that RGS1 specifically regulates other GPCR-mediated pathways that were not tested in this study. In contrast to *Rgs1* and *Rgs2* deficient mice being hypertensive, contradictory observations have been found in *Rgs5*^*−/−*^ mice. *Rgs5*^*−/−*^ mice have been shown to be hypotensive [29] and hypertensive [30]. The mechanism for hypotension is thought to occur through inhibition of NO-mediated dilatory signalling. However, in arterial VSMCs, RGS5 inhibits Ang II and Endothelin-1-induced intracellular Ca^2+^ transients mediated by Gαi and Gαq signalling [31]. These contrasting roles imply that RGS proteins function in a complex system of GPCR signal transduction by their selectivity towards specific G-proteins. In line with the findings observed for Rgs1 in this study, Rgs2 and Rgs5 mRNA transcripts are also regulated by Ang II in VSMCs [32] and aortas from Ang II infused mice [30], respectively. The in vivo Ang II infusion model indicated Rgs1 provided negative feedback to Ang II signalling and provided the opportunity to investigate the prolonged Ang II treatment of endothelial cells and VSMCs which showed down-regulation of Rgs1. Together, these data suggest that Rgs1, Rgs2 and Rgs5 are functionally important negative regulators of Ang II signalling in the vessel wall.

In addition to the alteration in the contractile response, a blunted endothelial dependent dilator response was also observed in both aorta and resistance mesenteric arteries from *Rgs1*^*−/−*^*ApoE*^*−/−*^ mice. Both ACh and the PAR-2 agonist SLIGRL act via Gq11 dependent endothelial cell receptors. If RGS1 were coupled to these GPCRs then it would be expected that loss of RGS1 would result in an enhanced dilator response. In our study, a significant proportion of the dilator response to Ach was inhibited by NOS inhibition, indicating that the majority of the dilatory response was due to NOS derived vasodilators. Previous in vivo studies have shown that a slow pressor dose of Ang II is associated with a significant increase in oxidative stress [33]. The blunted dilatory response observed in *Rgs1*^*−/−*^*ApoE*^*−/−*^ mice could be due to chronic over activation of the Ang II signalling pathway leading to increased reactive oxygen species and decreased NO bioavailability. Although we did not observed any differences in hydrogen peroxide production in VSMCs stimulated with Ang II, this does not account for possible changes in vascular tissue. Interestingly, in contrast to the blunted response to dilation observed with Ach, the dilatory response to the PAR-2 agonist SLIGRL did not show a reduction in the maximum dilatory response but instead a decreased sensitivity. This difference could be due to the relative potency of these two compounds as SLIGRL has been shown to induce a more potent dilatory response compared to Ach [34]. However, it could also be due to differences in the relative contribution of vasodilators; SLIGRL has been shown to utilize both NOS-cGMP dependent and independent mechanisms [35].

In conclusion, our results indicate that RGS1 regulates blood pressure to a significant extent to relax the resistance vasculature and attenuate vasoconstrictor signalling in the vasculature. We propose that RGS1 terminates Ang II signalling through accelerating the inactivation of the Gaq subunit of AT1 receptors that can propagate signals via multiple pathways including downstream Erk1/2 signalling pathways and intracellular calcium levels, which results in attenuated VSMC contraction. Furthermore, genetic impairment of Rgs1 therefore may contribute to the development of hypertension. These findings further promote the importance of RGS proteins as potential targets for the treatment of hypertension. Specifically, pharmacological agents that up-regulate RGS1 expression or activity in the vessel wall may augment the action of vasodilatory agonists and provide a novel means of treating hypertension.

The following is the supplementary data related to this article.

Supplementary Fig. 1 Flow cytometric analysis of FLAG intracellular expression. MOVAS cells were transfected with an empty vector control plasmid or FLAG tag Rgs1 plasmid and 48 hours later, intracellular staining using an anti-FLAG-FITC antibody was performed. The grey curve represents the empty vector control and the green curve represents the fluorescent intensity of the FLAG-FITC positive population. The population of Rgs1 transfected cells was 72 % FLAG positive at this time point. Supplementary Fig. 1
