# Supplementary material for: Vascular wall regulator of G-protein signalling-1 (RGS-1) is required for angiotensin II–mediated blood pressure control
Source: Vascul Pharmacol. 2018 Sep;108:15–22. doi: 10.1016/j.vph.2018.04.002 (PMC6073721; doi:10.1016/j.vph.2018.04.002)
Supplement: Supplementary Fig. 1 — Flow cytometric analysis of FLAG intracellular expression. MOVAS cells were transfected with an empty vector control plasmid or FLAG tag Rgs1 plasmid and 48 hours later, intracellular staining using an anti-FLAG-FITC antibody was performed. The grey curve represents the empty vector control and the green curve represents the fluorescent intensity of the FLAG-FITC positive population. The population of Rgs1 transfected cells was 72 % FLAG positive at this time point. [file mmc1.pptx]

## Slide 1
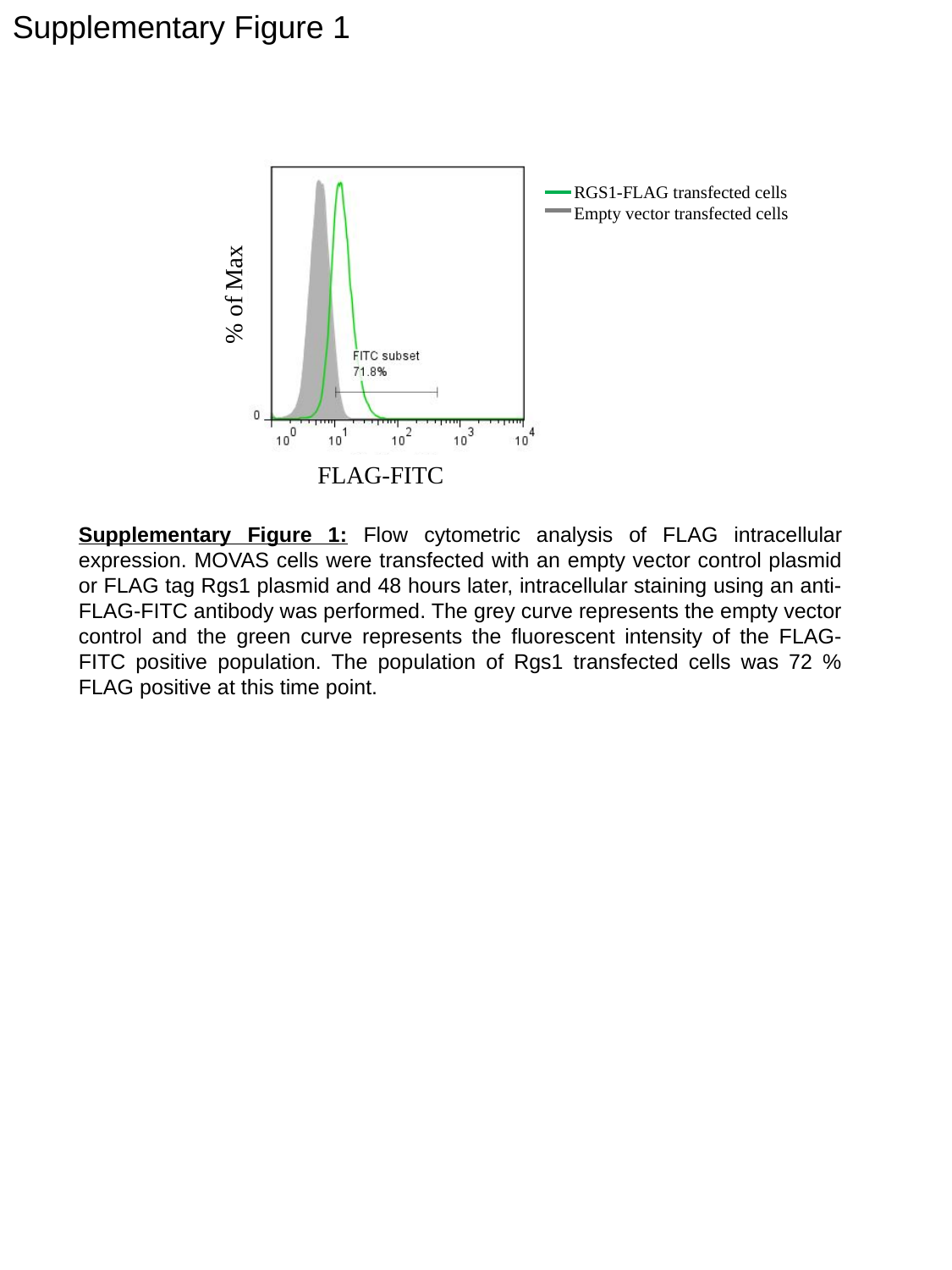

Supplementary Figure 1
RGS1-FLAG transfected cells
Empty vector transfected cells
% of Max
FLAG-FITC
Supplementary Figure 1: Flow cytometric analysis of FLAG intracellular expression. MOVAS cells were transfected with an empty vector control plasmid or FLAG tag Rgs1 plasmid and 48 hours later, intracellular staining using an anti-FLAG-FITC antibody was performed. The grey curve represents the empty vector control and the green curve represents the fluorescent intensity of the FLAG-FITC positive population. The population of Rgs1 transfected cells was 72 % FLAG positive at this time point.
